# Protein phosphorylation in plant immunity: insights into the regulation of pattern recognition receptor-mediated signaling

**DOI:** 10.3389/fpls.2012.00177

**Published:** 2012-08-03

**Authors:** Chang-Jin Park, Daniel F. Caddell, Pamela C. Ronald

**Affiliations:** Department of Plant Pathology and the Genome Center, University of California at Davis,Davis, CA, USA

**Keywords:** EFR, FLS2, pattern recognition receptor, plant immunity, post-translational modifications, protein phosphorylation, XA21

## Abstract

Plants are continuously challenged by pathogens including viruses, bacteria, and fungi. The plant immune system recognizes invading pathogens and responds by activating an immune response. These responses occur rapidly and often involve post-translational modifications (PTMs) within the proteome. Protein phosphorylation is a common and intensively studied form of these PTMs and regulates many plant processes including plant growth, development, and immunity. Most well-characterized pattern recognition receptors (PRRs), including *Xanthomonas* resistance 21, flagellin sensitive 2, and elongation factor-Tu receptor, possess intrinsic protein kinase activity and regulate downstream signaling through phosphorylation events. Here, we focus on the phosphorylation events of plant PRRs that play important roles in the immune response. We also discuss the role of phosphorylation in regulating mitogen-associated protein kinase cascades and transcription factors in plant immune signaling.

## INTRODUCTION

Proteins can undergo various post-translational modifications (PTMs) that affect their conformation, activity, stability, and localization. These PTMs, which are often reversible, are highly specific regulators of many cellular processes (Jensen, 2004). Currently, more than 300 types of PTMs have been described including ubiquitination, sumoylation, sulfation, glycosylation, and phosphorylation (Stulemeijer and Joosten, 2008; Ghelis, 2011). Phosphorylation is one of the most predominant PTMs and one-third of all eukaryotic proteins are thought to be phosphorylated (Olsen et al., 2006). Protein phosphorylation in eukaryotes predominantly occurs on serine (Ser) and threonine (Thr) residues, whereas phosphorylation on tyrosine (Tyr) residues is much less abundant (de la Fuente van Bentem and Hirt, 2009). Based on a recent large-scale phosphorylation study, the relative abundances of pSer, pThr, and pTyr were estimated to be 82.7, 13.1, and 4.2% in *Arabidopsis* and 84.8, 12.3, and 2.9% in rice (Sugiyama et al., 2008; Nakagami et al., 2010). Phosphorylation occurring on unusual residues such as histidine, lysine, and arginine (Besant and Attwood, 2005; Ciesla et al., 2011) will not be reviewed, because their involvement in plant immunity has not yet been elucidated.

A large body of evidence demonstrates that phosphorylation is essential for immune responses in animals and plants. For example, in animals, nearly 7,000 phosphorylation sites on more than 1,800 phosphoproteins were identified in response to lipopolysaccharide activation (Weintz et al., 2010). In *Arabidopsis*, more than 1,170 phosphopeptides from 472 phosphoproteins were identified after treatments with flg22 or xylanase, both of which elicit immune responses in *Arabidopsis* cell cultures (Benschop et al., 2007). These results indicate that many proteins are differentially phosphorylated and that the phosphorylation events are essential to both animal and plant immune responses. In this review, we focus primarily on phosphorylation events mediated by plant pattern recognition receptors (PRRs) that play important roles in the immune response.

## PATTERN RECOGNITION RECEPTORS IN RICE AND *ARABIDOPSIS*

Plant innate immunity is controlled by a set of defined receptors referred to as PRRs. A more detailed description of PRRs can be found in recent reviews (Ronald and Beutler, 2010; Schwessinger and Ronald, 2012). In general, recognition of conserved microbial signatures (also called pathogen-associated molecular patterns, PAMP) by PRRs triggers mitogen-associated protein kinase (MAPK) activation, production of reactive oxygen species (ROS), Ca^2^^+^ burst, transcriptional reprogramming, hormone biosynthesis, and deposition of callose in the cell wall (Nurnberger et al., 2004; Ronald and Beutler, 2010; Segonzac and Zipfel, 2011).

**FIGURE 1:**
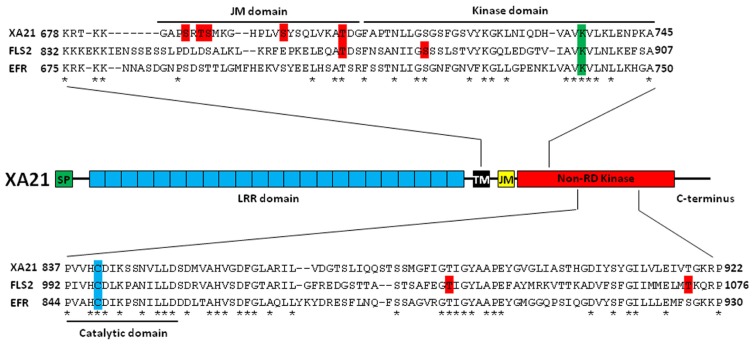
**Characterized Ser/Thr residues of pattern recognition receptors in plants**.*Top*: Identified and proposed autophosphorylation sites on rice XA21 and *Arabidopsis* FLS2 and EFR are highlighted in red. The conserved lysine that is essential for autophosphorylation is highlighted in green. The JM, kinase, and catalytic domains are indicated by black brackets. *Center*: The domain structure of rice XA21. *Bottom*: Alignment of the catalytic domains of XA21, FLS2, and EFR. The cysteine that replaces the R in these non-RD kinases is highlighted in blue. Putative autophosphorylation sites of FLS2 are highlighted in red. Amino acids that are conserved between XA21, FLS2, and EFR are marked as “*”. SP, signal peptide; LRR domain, 23 leucine-rich repeats domain; TM, transmembrane domain; JM, juxtamembrane domain; non-RD kinase, non-arginine–aspartate kinase; XA21, rice *Xanthomonas* resistance 21; FLS2, *Arabidopsis* flagellin sensitive 2; EFR, *Arabidopsis* elongation factor-Tu receptor.

The rice PRR, *Xanthomonas* resistance 21 (XA21), recognizes a conserved sulfated peptide called AxY^S^22, derived from the *Xanthomonas oryzae* pv. *oryzae* (*Xoo*) protein Ax21 (activator of XA21-mediated immunity; Lee et al., 2009). In *Arabidopsis*, two additional plant PRRs have been well-characterized. These are flagellin sensitive 2 (FLS2) and elongation factor (EF)-Tu receptor (EFR), which recognize the flg22 peptide from flagellated bacteria and the EF-Tu-derived peptide elf18, respectively (Gomez-Gomez and Boller, 2000; Zipfel et al., 2006). These PRRs consist of an extracellular leucine-rich repeat (LRR) domain, a transmembrane (TM) domain, a juxtamembrane (JM) domain, and an intracellular non-arginine–aspartate (non-RD) kinase domain (**Figure 1**; Dardick and Ronald, 2006; Schwessinger and Ronald, 2012).

Non-RD kinases typically carry a cysteine (C), or glycine (G) before the conserved catalytic aspartate (D) residue. All plant receptor kinases (RKs) characterized to date that carry the non-RD kinase motif are involved in recognition of conserved microbial signatures (Schwessinger and Ronald, 2012). In contrast, the larger group of RD kinases have an arginine (R) immediately preceding the conserved catalytic aspartate (D). RD kinases are known to perform more diverse functions and are often associated with developmental processes. RD kinases also work in partnership with non-RD kinases to transduce immune responses. In *Arabidopsis*, brassinosteroid insensitive 1 (BRI1)-associated kinase 1 (BAK1), an RD kinase, was initially identified as a positive regulator of brassinosteroid responses. BAK1 forms an *in vivo* ligand-dependent complex with the BRI1 receptor (Li et al., 2002; Nam and Li, 2002). Further research revealed that BAK1 is also involved in PRR-mediated signaling, physically interacting with the non-RD kinases FLS2 and EFR (Chinchilla et al., 2007,^2009^; Schwessinger et al., 2011). BAK1 null mutants are compromised in their responsiveness to several other conserved microbial signatures including HrpZ (hypersensitive response and pathogenicity Z), lipopolysaccharides, and peptidoglycans (Heese et al., 2007; Shan et al., 2008). The rice ortholog of BAK1, XA21-associated kinase 1 (XAK1), is required for XA21-mediated immunity (Chen et al., unpublished). These results demonstrate that PRRs utilize coregulatory receptors carrying RD kinases as signaling partners to transduce the immune response.

## PHOSPHORYLATIONS OF PATTERN RECOGNITION RECEPTORS

In accordance with an essential role of phosphorylation in immune signaling, phosphorylation of FLS2 is the first step in the FLS2-mediated intracellular signaling events (Boller and Felix, 2009). *De novo* phosphorylation of a FLS2/BAK1 complex is clearly detectable in cells 15 s after the addition of flg22 using *in vivo* labeling with short pulses of [^33^P]orthophosphate (Schulze et al., 2010). Treatment with protein kinase inhibitors is able to block a broad spectrum of early defense responses (Lecourieux et al., 2002; Navazio et al., 2002; Kadota et al., 2004).

**FIGURE 2:**
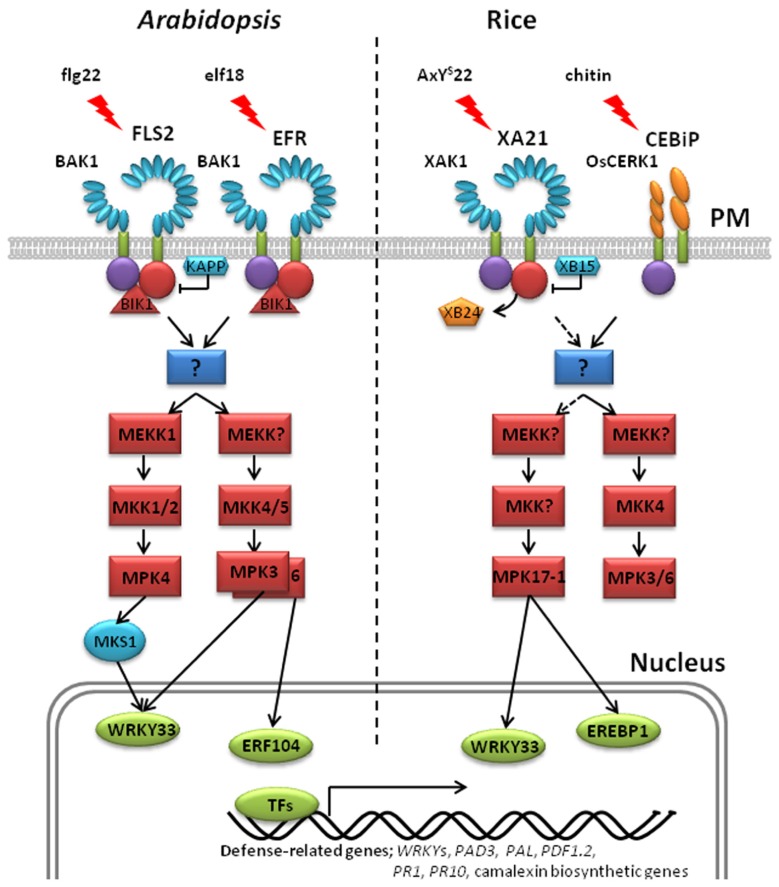
**Models for pattern recognition receptor-mediated phosphorylation pathways in *Arabidopsis* and rice**. *Left*: *Arabidopsis* FLS2 and BAK1 associate with the membrane-associated cytoplasmic kinase *Botrytis*-induced kinase 1 (BIK1) *in vitro* and *in vivo* (Lu et al., 2010). In the resting state, *Arabidopsis* FLS2 interacts with BIK1. Flg22 perception induces FLS2 and BAK1 association and phosphorylation. Activated BAK1 phosphorylates BIK1, which in turn transphosphorylates the FLS2/BAK1 complex. Phosphorylated BIK1 is released from the FLS2/BAK1 complex to activate downstream intracellular signaling. No direct phosphorylation targets of FLS2 have yet been identified. At least two MAPK cascades are initiated downstream of activated FLS2, leading to the phosphorylation of the adaptor protein MKS1 and the transcription factors, AtWRKY33 and ERF104. Kinase-associated protein phosphatase (KAPP), a PP2C, blocks the activated FLS2 signaling and attenuates the downstream immune response. EFR-mediated immunity is believed to trigger the same MAPK cascades as FLS2. *Right*: In the resting state,**rice XA21 forms an *in vivo* complex with the ATPase, XB24, and the XA21-associated kinase, XAK1. Association of XAK1 and XA21 requires the XA21 JM and kinase domains. Binding of AxY^S^22 to XA21 induces dissociation of XA21 from XB24 and activates XA21, triggering autophosphorylation. Activated XA21 likely activates a MAPK cascade that includes MPK17-1, leading to the phosphorylation of the transcription factors, OsWRKY33 and OsEREBP1. Recruitment of XB15 to the XA21 JM domain and subsequent dephosphorylation of phosphorylated residue(s) attenuates XA21 signaling. Cleavage of XA21 and translocalization of the intracellular kinase domain to the nucleus is required for the XA21-mediated immune response (Park and Ronald, 2012). Unlike other well-characterized PRRs, the rice receptors CEBiP and OsCERK1 contain extracellular LysM (lysine motif) domains in place of LRR domains (Kaku et al., 2006; Shimizu et al., 2010). Upon chitin perception, the CEBiP and OsCERK1 complex activates MAPK cascades. In *Arabidopsis* and rice, phosphorylation of transcription factors leads to large-scale transcriptional reprogramming, including the activation of *WRKY*s, *PAD3*, *PAL*, *PR*s, *PDF1.2*, and camalexin biosynthetic genes.

In animals, signal transduction is often regulated by phosphorylation of residues in the JM domain of RKs (Aifa et al., 2006; Thiel and Carpenter, 2007). It is now becoming clear that plant PRRs, at least XA21 and FLS2, are also phosphorylated on residues in their JM domains (**Figure 1**; **Table 1**). Targeted mutagenesis of the XA21 JM domain indicated that amino acids Ser^686^, Thr^688^, and Ser^689^ are autophosphorylated and required to maintain XA21 protein stability (Xu et al., 2006). Transgenic rice carrying XA21 mutants with alanine replacement of these three sites display partially compromised resistance compared to wildtype XA21 plants (Xu et al., 2006). Thr^705^ in the XA21 JM domain is also an important phosphorylation site and also affects the autophosphorylation activity of XA21 (Chen et al., 2010b). The XA21 mutant derivatives, XA21^T705A^ and XA21^T705E^, are both unable to transduce the XA21-mediated immune response. The importance of the JM domain in XA21-mediated immunity was also demonstrated through isolation of XA21-binding proteins (XBs). For example, the protein phosphatase 2C XB15 no longer interacts with XA21^S697A^, indicating that Ser697 in the JM domain is critical for interaction with XB15 (Park et al., 2008). Autophosphorylated XA21 is dephosphorylated by XB15 *in vitro*, suggesting that the function of XB15 is to attenuate the XA21-mediated innate immune response. The ATPase XB24 also associates with the XA21 JM domain and uses ATP to promote phosphorylation of certain Ser/Thr sites on XA21, keeping the XA21 protein in an inactive state. Upon recognition of sulfated Ax21, the XA21 kinase disassociates from XB24 and is activated, triggering downstream defense responses (Chen et al., 2010c; **Figure 2**).

**Table 1:** Summary of rice and *Arabidopsis*PRRs.

Organism	PRR	PRR class	Phosphorylation site	Ligand	Interacting protein	Protein class
Rice	XA21	LRR RK, non-RD	S686^1^	Ax21	XAK1^4^	LRR RK
		kinase	T688^1^	(AxY^S^22)^3^	XB3^5^	E3 ubiquitin ligase
			S689^1^		XB10^6^	WRKY transcription factor
			T705^2^		XB15^7^	Protein phosphatase 2C
					XB24^8^	ATPase
					ROX1^9^	Thiamine pyrophosphokinase
					ROX2^9^	NOL1/NOP2/sun protein
	CEBiP	LysM	NA	Chitin oligosaccharide^10^	OsCERK1^11^^,^^12^	LysM RK, RD kinase
*Arabidopsis*	FLS2	LRR RK, non-RD	T867^13^	Flagellin	BAK11^15^	LRR RK
		kinase	S878^13^	(flg22)^14^	BIK1^16^	Cytoplasmic kinase
			T1040^13^		KAPP^17^	Protein phosphatase 2C
			T1072^13^		BKK1, SERK1, SERK2^18^	LRR RK
					PUB12, PUB13^19^	E3 ubiquitin ligase
					SCD1^20^^,^^21^	DENN domain
					ACA8^22^	Calcium ATPase
	EFR	LRR RK, non-RD	NA	Elongation factor-Tu (elf18)^23^	BAK1^15^	LRR RK
		kinase			BIK1, PBL1^16^^,^^24^	Cytoplasmic kinase
					BKK1, SERK1, SERK2^18^	LRR RK
					SCD1^20^^,^^21^	DENN domain

*NA, not available; ACA, autoinhibited Ca^2+^-ATPase; BIK, Botrytis-induced kinase; BKK, BAK1-like kinase; NOL/NOP, nucleolar protein; CERK, chitin elicitor receptor kinase; PBL, PBS1-like; PUB, plant U-box; ROX, regulator of XA21; SCD, stomatal cytokinesis-defective; SERK, somatic-embryogenesis receptor-like kinase; SUN, Sad1-UNC-84 homology.*

*References:*

1 Xu et al. (2006),

2 Chen et al. (2010b),

3 Lee et al. (2009),

4 Chen et al., unpublished,

5 Wang et al. (2006),

6 Peng et al. (2008),

7 Park et al. (2008),

8 Chen et al. (2010c),

9 Lee et al. (2011),

10 Kaku et al. (2006),

11 Shimizu et al. (2010),

12 Schwessinger and Ronald (2012),

13 Robatzek et al. (2006),

14 Chinchilla et al. (2006),

15 Chinchilla et al. (2007),

16 Lu et al. (2010),

17 Gomez-Gomez et al. (2001),

18 Roux et al. (2011),

19 Lu et al. (2011),

20 Korasick et al. (2010),

21 Monaghan and Zipfel (2012),

22 Frei Dit Frey et al. (2012),

23 Zipfel et al. (2006), and

24 Zhang et al. (2010).

In *Arabidopsis*, the FLS2 JM residue Thr^867^ appears to be analogous to Thr^705^ in XA21 (**Figure 1**; **Table 1**; Chen et al., 2010b) and is also essential for the function of FLS2 (Robatzek et al., 2006). FLS2^T867V^ inhibits FLS2 internalization and response to flg22, indicating that both processes are intimately connected (Robatzek et al., 2006). Although the FLS2^T867V^ mutation had no effect on flg22-binding, FLS2^T867V^ mutant lines were insensitive to flg22 and displayed an enhanced disease susceptibility phenotype when challenged with pathogenic *Pseudomonas syringae*. Microscopic analysis of transgenic plants expressing FLS2^T867V^-GFP showed normal cell membrane localization of the mutant FLS2 protein. However, FLS2^T867V^ endocytosis is strongly reduced after flg22 treatment, suggesting that phosphorylation of FLS2^T867^ plays an important role in endocytosis. Further study is needed to determine if Thr^867^ of FLS2 is essential for FLS2 autophosphorylation in *Arabidopsis* and if Thr^70^^5^ of XA21 is critical for XA21 endocytosis in rice.

Four FLS2 amino acids were shown to be critical to FLS2 function using site-directed mutagenesis. Seedling growth of *Arabidopsis* transgenic lines expressing FLS2^T867V^, FLS2^T1040A^, FLS2^S878A^, and FLS2^T1072A^ were inhibited by flg22 treatment. Three of these mutations (FLS2^T867V^, FLS2^T1040A^, and FLS2^T1072A^) also abolished flg22-induced generation of ROS (Robatzek et al., 2006). It is not known if these sites are phosphorylated or if they are required for kinase activity.

In all protein kinases, it is well known that a conserved lysine residue is responsible for a phosphotransfer reaction (Carrera et al., 1993). The importance of this lysine for kinase function has been demonstrated for plant PRRs. For example, the Lys^736^ residue inside the XA21 kinase domain is essential for XA21 autophosphorylation (Liu et al., 2002). However, although catalytic activity of XA21 is essential for full resistance levels, the catalytically impaired XA21 mutant maintains partial resistance activity (Andaya and Ronald, 2003). The partial resistance is comparable to that of transgenic lines expressing XA21D, an XA21 family member consisting of an LRR domain but lacking a kinase domain, indicating that XA21 catalytic activity is not absolutely required for function. In *Arabidopsis*, a mutation in Lys^898^ of FLS2, which is analogous to Lys^736^ in XA21, abolishes MPK3 and MPK6 activation by flg22 when transiently overexpressed in protoplasts (Asai et al., 2002). Similarly, a kinase inactive mutation at Lys^741^ of EFR is unable to confer elf18-triggered ROS burst when transiently expressed in *Nicotiana benthamiana* (Schwessinger et al., 2011).

## MITOGEN-ACTIVATED PROTEIN KINASES SERVE AS INTERNODES IN PRR-MEDIATED IMMUNITY

Mitogen-associated protein kinase cascades are important for transmitting signals generated by receptors into cellular responses. Multiple studies support central roles for MAPK cascades in the immunity of *Arabidopsis*, parsley, tobacco, tomato, and rice (Frye et al., 2001; Zhang and Klessig, 2001; Cardinale et al., 2002; del Pozo et al., 2004; Pitzschke et al., 2009; Jung et al., 2010). Generally, MAP kinase kinase kinases (MAP3Ks, also called MEKKs) are activated by RKs. MAP3Ks activate downstream MAP kinase kinases (MAP2Ks, also called MKKs or MEKs) that in turn activate MAPKs (also called MPKs). MAPKs then target various proteins, which include other kinases, enzymes, and transcription factors (Khokhlatchev et al., 1998; Rodriguez et al., 2010). Genome-sequencing of *Arabidopsis* and rice have revealed the existence of approximately 60 MAP3Ks, 10 MAP2Ks, and 20 MAPKs in *Arabidopsis* (Group, 2002) and at least 75 MAP3Ks, 8 MAP2Ks, and 17 MAPKs in rice (Reyna and Yang, 2006; Rao et al., 2010).

In *Arabidopsis*, many studies have shown that activated FLS2 triggers MAPK signaling cascades (Asai et al., 2002; Chinchilla et al., 2007; Pitzschke et al., 2009; **Figure 2**). Initially, MEKK1 activates MPK4 which was previously shown to negatively regulate the defense response (Andreasson et al., 2005). At the MAPK kinase level, flg22-induced activation of MPK3/4/6 is dependent on MKK1/2, while MPK3 and MPK6 are also activated by MKK4 (Meszaros et al., 2006; Gao et al., 2008; Qiu et al., 2008b). Thus, two simultaneous MAPK cascades are postulated. The first consists of an unknown MEKK–MKK4/5–MPK3/6 and acts positively on FLS2-mediated signaling. The other consists of MEKK1–MKK1/2–MPK4 and acts negatively on the pathway (Nicaise et al., 2009). A physical interaction between MEKK1 and FLS2 has not been observed. Therefore, researchers are searching for signaling intermediates that function upstream of MEKK1 that would link FLS2 with the key MAPK cascades.

Elongation factor-Tu receptor-mediated signaling in *Arabidopsis* is thought to utilize a similar signal transduction pathway with FLS2. In-gel assays detect a rapid activation of MAPKs in EFR-mediated immune response after elf18 treatment (Zipfel et al., 2006). Treatment with both flg22 and elf18 at the same time induces the same MAPKs without an additive effect, indicating that these kinases belong to the same cellular pool of enzymes. These results suggest that PRR-mediated signaling induced by the two conserved microbial signatures, elf18 and flg22, converge at a step upstream of these kinases.

The role of MAPK cascades in PRR-mediated immunity has also been investigated in rice. For example, OsMPK3 [previously named OsBIMK1 (Song and Goodman, 2002)] and OsMPK17-1 [previously named OsBWMK1 (He et al., 1999)] both interact with XBs, suggesting that these MAP kinases are components of the XA21-mediated signaling pathway (Seo et al., 2011). OsMPK3 suppressing plants display enhanced resistance to *Xoo*, suggesting that it serves as negative regulator in the XA21-mediated response. In contrast, OsMPK17-1 knockouts displayed increased susceptibility to *Xoo*, suggesting a positive role in XA21-mediated immunity. OsMPK3/6 and OsMKK4 are activated by chitin (Kishi-Kaboshi et al., 2010; Kim et al., 2012).

## MAPKs PHOSPHORYLATE TRANSCRIPTION FACTORS

Transcriptional reprogramming of immune responses in the nucleus is regulated by transcription factors including the WRKY and ethylene-responsive factor [ERF, also called ethylene-responsive element binding protein (EREBP)] families (Gutterson and Reuber, 2004; Ishihama and Yoshioka, 2012). In animals, MAPKs are activated and then often translocate to the nucleus where MAPKs will directly or indirectly phosphorylate transcription factors (Harding et al., 2005; Rodriguez et al., 2010). Examples of nuclear localization of MAPKs have been reported in *Arabidopsis* and rice (Cheong et al., 2003; Yoo et al., 2008; Koo et al., 2009). Therefore, WRKY proteins and EREBPs constitute an important link between pathogen-activated MAPK signaling pathways and downstream transcriptional reprogramming.

High-density protein microarrays, employed to identify downstream factors of MAPKs in *Arabidopsis*, revealed that many WRKYs are directly regulated by MAPKs (Popescu et al., 2009). For example, *Arabidopsis* WRKY33 (AtWRKY33) is induced by conserved microbial signatures, such as the oomycete-derived peptide Pep25 (Lippok et al., 2007). Subsequent experiments showed that AtWRKY33 is phosphorylated by MPK3/MPK6 *in vivo* in response to *Botrytis cinerea* infection and by MPK4 at least *in vitro* (Mao et al., 2011). Phosphorylation of AtWRKY33 inhibits the growth of pathogenic fungi and bacteria by promoting the production of camalexin, a major antimicrobial phytoalexin. Mutation of MPK3/MPK6 phosphorylation sites in AtWRKY33 compromises its ability to complement the camalexin induction in the AtWRKY mutant. Another transcription factor, ethylene response factor (ERF104), is directly associated and phosphorylated by MPK6 but not MPK3 (Bethke et al., 2009). Perception of flg22 via FLS2 induces disruption of the MPK6/ERF104 complex, releasing ERF104 to its target promoters including *PDF1.2* (plant defensin 1.2).

There is an increasing body of evidence that suggests MAPKs also regulate transcription factors indirectly. Two WRKY transcription factors AtWRKY25 and AtWRKY33 interact with MPK4 substrate 1 (MKS1) in yeast, suggesting that these WRKYs regulate gene expression downstream of MPK4 (Andreasson et al., 2005). It was later reported that AtWRKY33 also forms an *in vivo* complex with MPK4 and MKS1 (Qiu et al., 2008a). However, although MKS1 is directly associated with AtWRKY33 and is phosphorylated by MPK4, no interaction has been detected between AtWRKY33 and MPK4 (Andreasson et al., 2005; Qiu et al., 2008a). This suggests MPK4 and AtWRKY33 associate indirectly and require the adaptor protein MKS1 for their interaction (Qiu et al., 2008a). Following pathogen perception, the MKS1–AtWRKY33 complex binds the phytoalexin deficient 3 (PAD3) promoter, which promotes camalexin synthesis.

To date, there are only a few reports suggesting that MAPKs phosphorylate rice transcription factors in response to pathogen infection. For example, OsMPK17-1 phosphorylates OsWRKY33 *in vitro*, which binds to the W-box element in the *OsPR1* gene promoter (Koo et al., 2009). OsMPK17-1 also phosphorylates the transcription factor OsEREBP1 *in vitro* (Cheong et al., 2003). Transient co-expression of OsMPK17-1 and OsEREBP1 in *Arabidopsis* protoplasts elevates the expression of the β-glucuronidase reporter gene driven by the ethylene-responsive element GCC box in several basic PR gene promoters. Thus, OsMPK17-1 is involved in rice defense signal transduction and is responsible for the direct phosphorylation of a transcription factor(s).

Although a role for MAPK-mediated phosphorylation of WRKYs has not been demonstrated for XA21-mediated immunity, several WRKYs interact directly with XA21 in yeast. For example, OsWRKY62, identified as XB10 in a yeast two-hybrid screen using the XA21 intracellular domain as bait, interacts with the XA21 kinase domain in rice protoplasts (Park and Ronald, 2012) and negatively regulates XA21-mediated immunity (Peng et al., 2008). Transgenic rice plants overexpressing OsWRKY62 are compromised in XA21-mediated immunity and are impaired in the activation the defense-related genes *OsPR1* and *OsPR10* (Peng et al., 2008). Additionally, OsWRKY76 was recently shown to negatively regulate XA21-mediated immunity when challenged with *Xoo* (Seo et al., 2011). Although these studies indicate a functional link between OsWRKYs and XA21-mediated immunity, XA21 has not been shown to directly phosphorylate the WRKYs, thus the role of phosphorylation is unknown.

## CONCLUSION AND PERSPECTIVES

Recognition of conserved microbial signatures by PRRs is critical to plant survival. PRR activation induces rapid autophosphorylation, leading to phosphorylation of many other proteins. Despite the importance of phosphorylation in PRR-mediated immunity, only a few phosphorylation sites of PRRs have been identified. Those phosphorylation sites were initially found by targeted mutagenesis. Although recent advances in phosphoproteomic analyses using mass spectrometry have greatly expanded our capability to identify phosphopeptides (Benschop et al., 2007; Nuhse et al., 2007; Stulemeijer and Joosten, 2008; Kersten et al., 2009), this approach has not yet lead to the identification of additional *in vivo* PRR phosphosites. This lack of success may be due to the observed rapid endocytosis and/or degradation of PRRs following perception of conserved microbial signatures (Robatzek et al., 2006; Robatzek, 2007; Chen et al., 2010a), which likely serves as a barrier to identifying PRR phosphorylation sites using mass spectrometry. Progress in mass spectrometric technology to enhance sensitivity of detection of low abundance phosphopeptides is needed to overcome this limitation. Once identified, such sites can be confirmed using independent techniques such as immunoblotting with anti-phospho-specific antibodies and *in vivo* genetic studies.

In addition to slow progress in identifying residues phosphorylated on the PRR itself, other proteins that could serve as targets of PRR phosphorylation have not yet been identified. Therefore, there is still a gap in our understanding of how precisely PRRs are able to initiate early signaling events such as activation of MAPKs, a rapid calcium influx and an oxidative burst. To answer these fundamental questions, it will be essential to identify such target proteins and to determine how these proteins regulate downstream events. Phosphoproteomic comparison is one method that can identify proteins that become phosphorylated during PRR-mediated immunity. For example, quantitative phosphoproteomic analyses performed on flg22- or xylanase-treated *Arabidopsis* cells successfully revealed several differentially phosphorylated proteins such as auxin efflux carriers and respiratory burst oxidase protein D (Nuhse et al., 2007; Stulemeijer and Joosten, 2008).

Another important goal is to identify the substrates of MAPKs that are phosphorylated during PRR-mediated immunity. To date, only a few transcription factors have been shown to be phosphorylated by MAPKs during PRR-mediated immune responses. Studies utilizing protein microarrays, protein complex immunoprecipitations, and phosphoproteomic analyses will continue to uncover additional transcription factors and other potential MAPK targets, further contributing to our understanding of the role of phosphorylation in plant immune responses.

## Conflict of Interest Statement

The authors declare that the research was conducted in the absence of any commercial or financial relationships that could be construed as a potential conflict of interest.
